# The Neuroendocrine Regulation of Food Intake in Fish: A Review of Current Knowledge

**DOI:** 10.3389/fnins.2016.00540

**Published:** 2016-11-29

**Authors:** Helene Volkoff

**Affiliations:** Departments of Biology and Biochemistry, Memorial University of NewfoundlandSt. John's, NL, Canada

**Keywords:** fish, hormones, feeding, appetite, diversity, brain, intestine

## Abstract

Fish are the most diversified group of vertebrates and, although progress has been made in the past years, only relatively few fish species have been examined to date, with regards to the endocrine regulation of feeding in fish. In fish, as in mammals, feeding behavior is ultimately regulated by central effectors within feeding centers of the brain, which receive and process information from endocrine signals from both brain and peripheral tissues. Although basic endocrine mechanisms regulating feeding appear to be conserved among vertebrates, major physiological differences between fish and mammals and the diversity of fish, in particular in regard to feeding habits, digestive tract anatomy and physiology, suggest the existence of fish- and species-specific regulating mechanisms. This review provides an overview of hormones known to regulate food intake in fish, emphasizing on major hormones and the main fish groups studied to date.

## Introduction

Feeding is a complex behavior consisting of food ingestion itself as well as foraging or appetitive behaviors (which reflect motivation to consume food; Keen-Rhinehart et al., 2013; Woods and Begg, 2016). Feeding is ultimately regulated by central feeding centers of the brain, which receive and process information from endocrine signals from both brain and periphery. These signals consist of hormones that increase (e.g., orexin; neuropeptide Y-NPY) or inhibit, (e.g., cocaine and amphetamine regulated transcript-CART; proopiomelanocortin-POMC) feeding. Feeding centers are also influenced by metabolic and neural peripheral signals providing information on meal ingestion and nutritional status (Volkoff, 2006; Volkoff et al., 2009a,b; Rui, 2013; Sobrino Crespo et al., 2014).

Fish are the most diversified group of vertebrates, with 33,200 species identified to date (FishBase, 2016), the bony fish (teleosts) containing more than half of all vertebrate species (Nelson, 2006). However, only relatively few fish species have been examined to date, with regards to their physiology, in particular feeding. The large numbers of fish species, habitats, feeding habits and digestive tract anatomy and physiology, as well as the number of extrinsic and intrinsic factors affecting feeding behavior and physiology (Volkoff et al., 2009a; Hoskins and Volkoff, 2012) most probably result in complex species-specific feeding regulating mechanisms in fish, with a number of hormones and tissues involved.

Research on the endocrine regulation of feeding in fish has progressed in recent years. New fish appetite-regulating hormones and species other than traditional models (such as goldfish, salmon and zebrafish) are gradually being examined. In addition, traditional techniques such as brain lesions and injections and biochemical purification of peptides, although still useful and being used, have been complemented by new approaches such as gene expression studies, quantitative PCR, genomics (microarrays, RNA-seq), proteomics and metabolomics, transgenesis, gene knockout and silencing, and *in vitro* (cell and tissue culture, perifusion) studies.

The field of fish feeding endocrine physiology is evolving very rapidly and up-to-date reviews are often lacking. One of the first reviews on the endocrine regulation of feeding by R.E. Peter in 1979 (Peter, 1979) mostly focused on growth and growth hormone (GH) but predicted regions of the brain that might be responsible for feeding regulation in fish. In 1986, Matty's review described early data on the effects of GH, thyroid hormones, insulin, and gonadal steroids on feeding (Matty, 1986). Ten years later, Le Bail and Boeuf's review formulated hypotheses on mammalian hormones (e.g., leptin) that might putatively regulate feeding in fish (Le Bail and Boeuf, 1997). In the early twenty-first century, a number of reviews report recent advances on the field and include an increasing number of hormones (e.g., NPY, orexins, CART), some more comparative (Lin et al., 2000; de Pedro and Björnsson, 2001; Volkoff et al., 2005; Gorissen et al., 2006; Volkoff, 2011; Hoskins and Volkoff, 2012), some more focused on a single species (e.g., goldfish Matsuda, 2009; Matsuda et al., 2011a) or a particular group of fish (e.g., elasmobranchs Demski, 2012), some focused on growth (Won and Borski, 2013), and some on aquaculture and behavior (Papoutsoglou, 2012).

The purpose of this review is to provide an up-to-date, brief overview of the hormones regulating food intake in fish, emphasizing on recent studies, major brain hormones and the main fish groups studied thus far.

## Overview of regulation of food intake

In fish, as in mammals (Sobrino Crespo et al., 2014), feeding behavior is regulated by specific regions in the brain, the so-called feeding centers. Early pioneer studies using stimulation and lesion experiments in teleosts (reviewed in Peter, 1979) and elasmobranchs (reviewed in Demski, 2012) seemed to indicate that the hypothalamic area was involved in feeding and that the brain control of feeding in fish might use mechanisms similar to those in mammals. However, whereas in mammals, the feeding centers appear to be restricted to the hypothalamus, evidence indicates that they might be more widespread in fish brains (Cerda-Reverter and Canosa, 2009).

Feeding centers are under the influence of hormones produced by the brain and the periphery. Neurohormones secreted by the brain, in particular the hypothalamic area, regulate energy balance by inhibiting (anorexigenic factors) or stimulating (orexigenic factors) feeding. Peripheral chemical (e.g., glucose) or endocrine (e.g., gastrointestinal hormones) factors released in the blood cross the blood brain barrier and have a direct action on feeding centers. Peripheral sensory information (mechanical or endocrine) carried by the vagus nerve can also affect feeding centers, via innervation from the brainstem (Volkoff, 2011).

## Hormones involved in food intake

**Figure 1:**
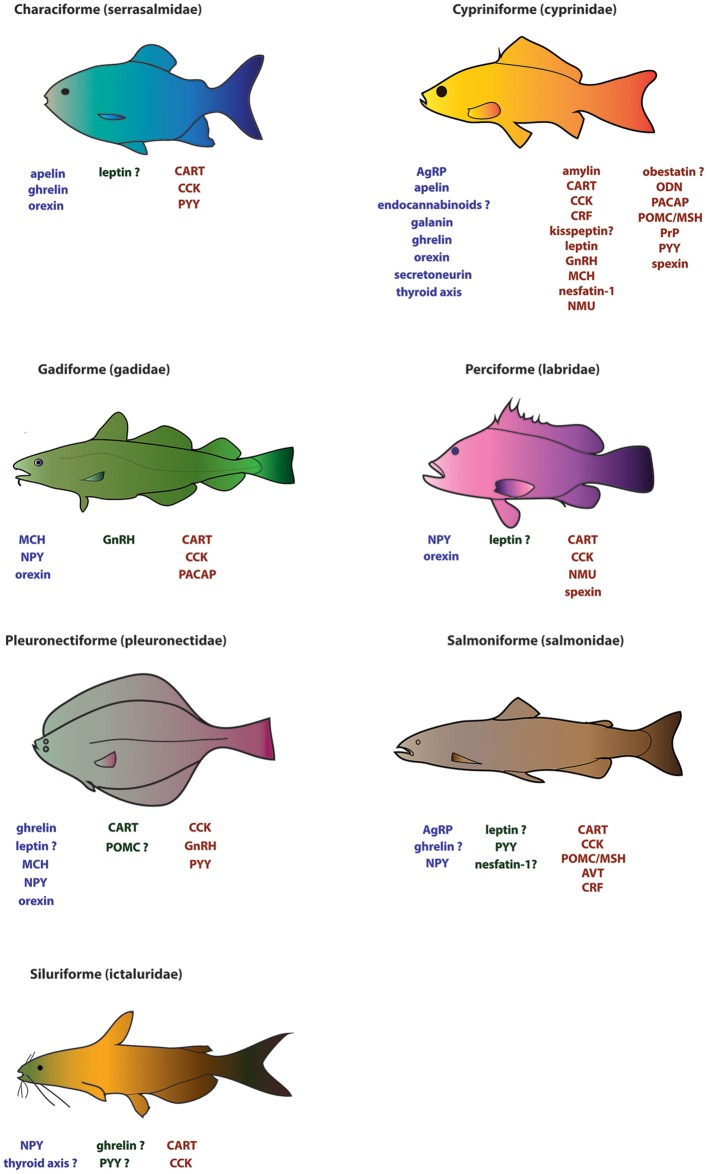
**Major appetite regulators known for seven of the most studied representative fish families (serrasalmidae, cyprinidae, gadidae, labridae, pleuronectidae, salmonidae, and ictaluridae)**. Factors in blue (far left) and in red (far right) under the fish diagrams represent putative orexigenic and anorexigenic factors, respectively. Factors in green (middle) represent factors with no established effect on feeding. A “?” indicates uncertainty with regards to the role of a given factor in regulating feeding.

The list of hormones regulating feeding in vertebrates is long and increasingly so. Here, focus will be placed on major hormones and newly examined appetite-regulating factors (but not on their receptors), and the phylogeny of the fish species examined to date. Table 1, Figure 1 summarize the hormones that have been examined in fish and their possible effects on feeding.

**Table 1:** **List of major hormones (in alphabetical order) potentially involved in the regulation of feeding in fish (by order, family and species studied)**.

**Hormone**	**Order**	**Family**	**Species**	**Major references**	**Effect on feeding**
AgRP	Cypriniforme	Cyprinidae	Common carp (*Cyprinus carpio*)	Zhong et al., 2013	+
			Common carp (*Cyprinus carpio*)	Wan et al., 2012	− ?
			Goldfish (*Carassius auratus)*	Cerdá-Reverter and Peter, 2003	+
			Ya fish (*Schizothorax prenanti*)	Wei et al., 2013	+
			Zebrafish (*Danio rerio)*	Song et al., 2003; Song and Cone, 2007	+
	Salmoniforme	Salmonidae	Atlantic salmon (*Salmo salar*)	Murashita et al., 2009a; Valen et al., 2011	−
			Arctic charr (*Salvelinus alpinus*)	Striberny et al., 2015	+
			Coho salmon (*Oncorhynchus kisutch)*	Kim et al., 2015	+
	Perciforme	Moronidae	Sea bass (*Dicentrarchus labrax*)	Agulleiro et al., 2014	+
Amylin	Cypriniforme	Cyprinidae	Goldfish (*Carassius auratus)*	Thavanathan and Volkoff, 2006	−
Apelin	Characiforme	Characidae	Blind cavefish (*Astyanax mexicanus)*	Penney and Volkoff, 2014	+
		Serrasalmidae	Red-bellied piranha (*Pygocentrus nattereri)*	Volkoff, 2014a	+
	Cyprinoforme	Cyprinidae	*Cyprinus carpio* and *Capoetta trutta*	Köprücü and Algül, 2015	?
			Goldfish (*Carassius auratus*)	Volkoff and Wyatt, 2009; Wong et al., 2013; Volkoff, 2014a; Zhang et al., 2016b	+
			Ya fish (*Schizothorax prenanti*)	Lin et al., 2014a	+
	Perciforme	Labridae	Cunner (*Tautogolabrus adspersus*)	Hayes and Volkoff, 2014	− ?
AVT (arginine vasotocin)	Salmoniforme	Salmonidae	Rainbow trout (*Oncorhynchus mykiss)*	Gesto et al., 2014	−
CART	Beloniformes	Adrianichthyidae	Medaka (*Oryzias latipes*)	Murashita and Kurokawa, 2011	−
	Characiforme	Characidae	Blind cavefish (*Astyanax mexicanus*)	Penney and Volkoff, 2014	−
			Dourado (*Salminus brasiliensis*)	Volkoff et al., 2016	0
		Serrasalmidae	Pacu (*Piaractus mesopotamicus*)	Volkoff et al., 2017	−
			Pirapitinga (*Piaractus brachypomus*)	Volkoff, 2015a	?
			Red-bellied piranha (*Pygocentrus nattereri*)	Volkoff, 2014a	−
	Cypriniforme	Cyprinidae	Common carp (*Cyprinus carpio*)	Wan et al., 2012	−
			Goldfish (*Carassius auratus*)	Abbott and Volkoff, 2011; Volkoff, 2012, 2014b; Zhang et al., 2016b	−
			Grass carp (*Ctenopharyngodon idellus*)	Zhou et al., 2013; Liu et al., 2014	−
			Zebrafish (*Danio rerio*)	Mukherjee et al., 2012; Nishio et al., 2012; Akash et al., 2014; Manuel et al., 2014, 2015; Libran-Perez et al., 2014; Woods et al., 2014; Guillot et al., 2016	−
	Gadiforme	Gadidae	Atlantic cod (*Gadus morhua*)	Kehoe and Volkoff, 2007	− ?
	Perciforme	Labridae	Cunner (Tautogolabrus adspersus)	Babichuk and Volkoff, 2013	−
	Pleuronectiformes	Pleuronectidae	Atlantic halibut (*Hippoglossus hippoglossus*)	Gomes et al., 2014	0
			Winter flounder (*Pseudopleuronectes americanus*)	MacDonald and Volkoff, 2009a	0
		Soleidae	Senegalese sole (*Solea senegalensis*)	Bonacic et al., 2015	−
	Rajiforme (elasmobranch)	Rajidae	Winter skate (*Raja ocellata*)	MacDonald and Volkoff, 2009b	0
	Salmoniforme	Salmonidae	Atlantic salmon (*Salmo salar*)	Murashita et al., 2009a; Burt et al., 2013; Kousoulaki et al., 2013	−
			Arctic Charr (*Salvelinus alpinus*)	Striberny et al., 2015	0
			Rainbow trout (*Oncorhynchus mykiss*)	Figueiredo-Silva et al., 2012; MacDonald et al., 2014	−
	Siluriforme	Clariidae	African sharptoothcatfish (*Clarias gariepinus*)	Subhedar et al., 2011	−
			Walking catfish (*Clarias batrachus*)	Barsagade et al., 2010	−
		Ictaluridae	Channel catfish (Ictalurus punctatus)	Kobayashi et al., 2008; Peterson et al., 2012	−
CCK	Characiforme	Characidae	Blind cavefish (Astyanax fasciatus mexicanus)	Wall and Volkoff, 2013	−
			Dourado (*Salminus brasiliensis)*	Pereira et al., 2015; Volkoff et al., 2016	−
			Thin dogfish (*Oligosarcus hepsetus)*	Vieira-Lopes et al., 2013	−
		Serrasalmidae	Pacu (*Piaractus mesopotamicus*)	Volkoff et al., 2017	−
			Pirapitinga (*Piaractus brachypomus)*	Volkoff, 2015a	−
			Red-bellied piranha (*Pygocentrus nattereri*)	Volkoff, 2014a	−
	Cypriniforme	Cyprinidae	Blunt snout bream (*Megalobrama amblycephala*)	Ping et al., 2013; Ji et al., 2015	−
			Common carp (*Cyprinus carpio*)	Zhong et al., 2013	−
			Goldfish (*Carassius auratus*)	Kang et al., 2010, 2011; Tinoco et al., 2015	−
			Grass carp (*Ctenopharyngodon idella*) juveniles	Liu et al., 2013	−
			Zebrafish (*Danio rerio*)	Koven and Schulte, 2012; Tian et al., 2015	−
	Gadiforme	Gadidae	Atlantic cod larvae (*Gadus morhua*)	Tillner et al., 2013	−
	Perciforme	Carangidae	Yellowtail (*Seriola quinqueradiata*)	Furutani et al., 2013; Hosomi et al., 2014	−
		Cichlidae	*Astatotilapia burtoni*	Grone et al., 2012	−
		Labridae	Cunner (*Tautogolabrus adspersus*)	Babichuk and Volkoff, 2013; Hayes and Volkoff, 2014	−
		Moronidae	Sea bass (*Dicentrarchus labrax*) larvae	Tillner et al., 2014	−
		Sciaenidae	Yellow croaker *(Larimichthys crocea*) larvae	Cai et al., 2015	−
		Sparidae	White sea bream (*Diplodus sargus)*	Micale et al., 2012	−
	Pleuronectiforme	Pleuronectidae	Atlantic halibut (*Hippoglossus hippoglossus*)	Kamisaka et al., 2001	−
			Olive flounder (*Paralichthys olivaceus*)	Kurokawa et al., 2000	−
			Winter flounder (*Pseudopleuronectes americanus*)	MacDonald and Volkoff, 2009a	−
	Salmoniforme	Salmonidae	Atlantic salmon (*Salmo salar*)	Valen et al., 2011	−
	Siluriforme	Ictaluridae	Channel catfish (*Ictalurus punctatus*)	Peterson et al., 2012	−
CRF/UCN system	Acipenseriformes	Acipenseridae	Siberian sturgeon (*Acipenser baerii*)	Zhang et al., 2016c	−
	Cypriniforme	Cyprinidae	Goldfish (*Carassius auratus*)	De Pedro et al., 1993	−
			Ya fish (*Schizothorax prenanti*)	Wang et al., 2014	−
	Salmoniforme	Salmonidae	Rainbow trout (*Oncorhynchus mykiss*)	Bernier and Craig, 2005; Ortega et al., 2013	−
Endocannabinoid system	Cypriniforme	Cyprinidae	Goldfish (*Carassius auratus*)	Cottone et al., 2013	+ ?
			Goldfish (*Carassius auratus)*	Cottone et al., 2009	+
	Perciforme	Sparidae	Sea bream (*Sparus aurata)*	Piccinetti et al., 2010	+
	Pleuronectiforme	Soleidae	Sole (*Solea solea*)	Palermo et al., 2013	?
Galanin	Cypriniforme	Cyprinidae	Goldfish (*Carassius auratus*)	de Pedro et al., 1995; Volkoff and Peter, 2001b; Unniappan et al., 2004	+
			Tench (*Tinca tinca*)	Guijarro et al., 1999	+
			Zebrafish (*Danio rerio*)	Li et al., 2013	+
Ghrelin	Anguilliformes	Anguillidae	Japanese eel (*Anguilla japonica*)	Lee et al., 2015	?
	Characiforme	Serrasalmidae	Pirapitinga (*Piaractus brachypomus*)	Volkoff, 2015a	?
			Red-bellied piranha (*Pygocentrus nattereri*)	Volkoff, 2015b	+
	Cypriniforme	Cyprinidae	Bunnei (*Barbus sharpeyi*)	Mabudi et al., 2011	?
			Gibel carp (*Carassius auratus gibelio*)	Zhou et al., 2016	+
			Goldfish (*Carassius auratus*)	Unniappan et al., 2002; Kang et al., 2011; Nisembaum et al., 2014; Blanco et al., 2016a,b	+
			Grass carp (*Ctenopharyngodon idella*)	Liu et al., 2014	+
			Zebrafish (*Danio rerio*)	Koven and Schulte, 2012	+
	Lepidosireniforme (dipnoid)	Protopteridae	West African lungfish (*Protopterus annectens*)	Kaiya et al., 2014	?
	Perciforme	Cichlidae	Tilapia (*Oreochromis mossambicus*)	Schwandt et al., 2010; Upton and Riley, 2013	+
		Scombridae	Pacific bluefin tuna (*Thunnus orientalis*)	Suda et al., 2012	?
	Pleuronectiforme	Pleuronectidae	Atlantic halibut (*Hippoglossus hippoglossus*)	Einarsdottir et al., 2011; Gomes et al., 2014	+
		Scophthalmidae	Juvenile turbot (*Scophthalmus maximus*)	Song et al., 2015	+
	Salmoniforme	Salmonidae	Atlantic salmon (*Salmo salar*)	Hevrøy et al., 2011; Vikesa et al., 2015	?
			Brown trout (*Salmo trutta*)	Tinoco et al., 2014a	+ ?
			Coho salmon (*Oncorhynchus kisutch)*	Kim et al., 2015	+
			Rainbow trout (*Oncorhynchus mykiss*)	Velasco et al., 2016	?
			Rainbow trout (*Oncorhynchus mykiss*)	Jönsson et al., 2010	−
	Siluriforme	Bagridae	Yellow catfish (*Pelteobagrus fulvidraco)*	Zhang et al., 2016a	+
		Ictaluridae	Channel catfish (*Ictalurus punctatus*)	Peterson et al., 2012	0
GnRH	Cypriniforme	Cyprinidae	Goldfish (*Carassius auratus*)	Hoskins et al., 2008; Matsuda et al., 2008	−
			Ya fish (*Schizothorax prenanti*)	Wang et al., 2014	−
			Zebrafish (*Danio rerio*)	Nishiguchi et al., 2012	−
	Gadiforme	Gadidae	Atlantic cod (*Gadus morhua*)	Tuziak and Volkoff, 2013a	0
	Pleuronectiforme	Pleuronectidae	Winter flounder (*Pseudopleuronectes americanus*)	Tuziak and Volkoff, 2013b	−
Kisspeptin	Cypriniforme	Cyprinidae	Goldfish (*Carassius auratus*)	Mawhinney, 2007	0
	Perciforme	Moronidae	Sea bass (*Dicentrarchus labrax*)	Escobar et al., 2016	+ ?
Leptin	Beloniforme	Adrianichthyidae	Medaka (*Oryzias latipes*)	Chisada et al., 2014	−
	Characiforme	Serrasalmidae	Pacu (*Piaractus mesopotamicus*)	Volkoff et al., 2017	0 ?
			Pirapitinga (*Piaractus brachypomus*)	Volkoff, 2015a	?
			Red-bellied piranha (*Pygocentrus nattereri*)	Volkoff, 2015b	0 ?
	Cypriniforme	Cyprinidae	Blunt snout bream *Megalobrama amblycephala*	Xu et al., 2016	−
			Common carp (Cyprinus carpio)	Bernier et al., 2012	−
			Goldfish (*Carassius auratus*)	Volkoff et al., 2003; Vivas et al., 2011; Tinoco et al., 2012, 2014b; Yan et al., 2016	−
			Grass carp (*Ctenopharyngodon idellus)*	Lu et al., 2015; Li et al., 2010	−
			Grass carp (*Ctenopharyngodon idellus*)	Li A. et al., 2016	?
			Topmouth culter (*Culter alburnus*)	Wang et al., 2013	?
			White-clouds minnow (Tanichthys albonubes)	Chen et al., 2016b	−
			Zebrafish (*Danio rerio*)	Tian et al., 2015; Cui et al., 2016; Michel et al., 2016	−
	Gadiforme	Lotidae	Burbot (*Lota lota*)	Nieminen et al., 2003	+ ?
	Gymnotiforme	Sternopygidae	Electric fish (*Eigenmannia virescens*)	Sinnett and Markham, 2015	?
	Perciforme	Carangidae	Golden pompano (*Trachinotus blochii*)	Wu et al., 2016	?
		Centrarchidae	Green sunfish (*Lepomis cyanellus*)	Johnson et al., 2000	+ ?
		Cichlidae	Tilapia (*Oreochromis mossambicus*)	Baltzegar et al., 2014; Douros et al., 2014	?
			Nile tilapia (*Oreochromis niloticus*)	Shpilman et al., 2014	?
		Labridae	Orange-spotted grouper (*Epinephelus coioides*)	Huang et al., 2014	?
		Labridae	Orange-spotted grouper (*Epinephelus coioides*)	Zhang et al., 2013	?
		Moronidae	European sea bass (*Dicentrarchus labrax*)	Gambardella et al., 2012	?
			Striped bass (*Morone saxatilis*)	Won et al., 2012	−
		Percichthyidae	Mandarin fish (*Siniperca chuatsi*)	Yuan et al., 2016	?
			Murray cod (*Maccullochella peelii peelii*)	Ettore et al., 2012 Varricchio et al., 2012	?
		Scombridae	Mackerel (*Scomber japonicus*)	Ohga et al., 2015	?
	Pleuronectiformes	Paralichthyidae	Fine flounder (*Paralichthys adspersus*)	Fuentes et al., 2012	+
	Salmoniforme	Salmonidae	Atlantic salmon (*Salmo salar*)	Rønnestad et al., 2010; Murashita et al., 2011; Trombley et al., 2012, 2014; Kullgren et al., 2013; Moen and Finn, 2013	?
			Arctic charr (*Salvelinus alpinus*)	Froiland et al., 2012; Jørgensen et al., 2013	?
			Rainbow trout (*Oncorhynchus mykiss*)	Gong et al., 2016	−
			Rainbow trout (*Oncorhynchus mykiss*)	Varricchio et al., 2012; Francis et al., 2014; MacDonald et al., 2014; Johansson and Björnsson, 2015; Salmeron et al., 2015; Johansson et al., 2016; Pfundt et al., 2016	?
	Siluriforme	Bagridae	Yellow catfish (*Pelteobagrus fulvidraco*)	Gong et al., 2013; Song et al., 2015; Zheng et al., 2015	?
MCH	Beloniforme	Adrianichthyidae	Medaka (*Oryzias latipes*)	Qu et al., 1996	0
	Carcharhiniforme	Sphyrnidae	Scalloped hammerhead shark (*Sphyrna lewini*)	Mizusawa et al., 2012	0
	Characiforme	Serrasalmidae	Red-bellied piranha (*Pygocentrus nattereri*)	Pérez Sirkin et al., 2013	?
	Cypriniforme	Cyprinidae	Goldfish (*Carassius auratus*)	Shimakura et al., 2006; Matsuda et al., 2007a	−
			Ya fish (*Schizothorax prenanti*)	Wang et al., 2016	+
	Gadiforme	Gadidae	Atlantic cod (*Gadus morhua*)	Tuziak and Volkoff, 2013a; Tuziak et al., 2014	+
	Pleuronectiforme	Pleuronectidae	Barfin flounder (*Verasper moseri*)	Takahashi et al., 2004; Amiya et al., 2008	+
			Olive flounder (*Paralichthys olivaceus*)	Kang and Kim, 2013b	+
			Starry flounder (*Platichthys stellatus*)	Kang and Kim, 2013a	+
			Winter flounder (*Pseudopleuronectes americanus*)	Tuziak and Volkoff, 2012	+
Nesfatin-1	Cypriniforme	Cyprinidae	Goldfish (*Carassius auratus*)	Gonzalez et al., 2010; Kerbel and Unniappan, 2012; Blanco et al., 2016a; Sundarrajan et al., 2016	−
			Zebrafish (*Danio rerio*)	Hatef et al., 2015	−
			Ya fish (*Schizothorax prenanti*)	Lin et al., 2014b	−
	Salmoniforme	Salmonidae	Rainbow trout (*Oncorhynchus mykiss*)	Caldwell et al., 2014	0
Neuromedin S	Cypriniforme	Cyprinidae	Zebrafish (*Danio rerio*)	Chen et al., 2016a	+ ?
Neuropepide B	Perciforme	Cichlidae	Nile tilapia (*Oreochromis niloticus*)	Yang et al., 2014	+ ?
NMU	Cypriniforme	Cyprinidae	Common carp (*Cyprinus carpio*)	Kono et al., 2012	?
	Cypriniforme	Cyprinidae	Goldfish (*Carassius auratus*)	Maruyama et al., 2008, 2009	−
	Perciforme	Labridae	Orange-spotted grouper (*Epinephelus coioides*)	Li et al., 2015	−
NPY	Characiforme	Characidae	Dourado (*Salminus brasiliensis*)	Pereira et al., 2015	+ ?
	Chimaeriformes	Callorhinchidae	Elephant fish (*Callorhinchus milii*)	Larsson et al., 2009	+ ?
	Cypriniforme	Cyprinidae	Blunt snout bream (*Megalobrama amblycephala*)	Xu et al., 2016	+
			Goldfish (*Carassius auratus*)	Lopez-Patino et al., 1999; de Pedro et al., 2000; Narnaware et al., 2000; Hoskins and Volkoff, 2012	+
			Grass carp (*Ctenopharyngodon idellus*)	He et al., 2013; Zhou et al., 2013; Jin et al., 2015	+
			Jian carp (*Cyprinus carpio* var. Jian)	Tang et al., 2014	+
			Ya fish (*Schizothorax prenanti*)	Wei et al., 2014	+
			Zebrafish (*Danio rerio*)	Yokobori et al., 2012; Dalmolin et al., 2015	+
	Gadiforme	Gadidae	Atlantic cod (*Gadus morhua*)	Kehoe and Volkoff, 2007; Tuziak et al., 2014	+
			Atlantic cod (*Gadus morhua*)	Kortner et al., 2011	?
	Gonorynchiforme actinopterygii	Chanidae	Milkfish (*Chanos chanos*)	Lin et al., 2016	?
	Perciforme	Carangidae	Yellowtail (*Seriola quinqueradiata*)	Hosomi et al., 2014	+
		Cichlidae	*Astatotilapia burtoni*	Grone et al., 2012	+
		Labridae	Cunner (*Tautogolabrus adspersus*)	Babichuk and Volkoff, 2013	+
			Orange-spotted grouper (*Epinephelus coioides*)	Tang et al., 2013	+
		Moronidae	Sea bass (*Dicentrarchus labrax*)	Leal et al., 2013	+
		Percichthyidae	Mandarin fish (*Siniperca chuatsi*)	Sun et al., 2014	?
		Rachycentridae	Cobia (*Rachycentron canadum*)	Van Nguyen et al., 2013	+
		Osphronemidae	Snakeskin gourami (*Trichogaster pectoralis*)	Boonanuntanasarn et al., 2012	+
	Pleuronectiforme	Pleuronectidae	Olive flounder (*Paralichthys olivaceus*)	Wang et al., 2015	+
		Paralichthyidae	Brazilian flounder (*Paralichthys orbignyanus*)	Campos et al., 2012	+
	Salmoniforme	Salmonidae	Atlantic salmon (*Salmo salar*)	Silverstein et al., 1998; Valen et al., 2011; Figueiredo-Silva et al., 2012	+
	Salmoniforme	Salmonidae	Chinook salmon (*Oncorhynchus tshawytscha*)	Silverstein et al., 1998	+
		Salmonidae	Rainbow trout (*Oncorhynchus mykiss*)	Aldegunde and Mancebo, 2006	+
	Siluriformes	Ictaluridae	Channel catfish (*Ictalurus punctatus*)	Silverstein and Plysetskaya, 2000; Peterson et al., 2012; Schroeter et al., 2015	+
	Tetraodontiformes	Tetraodontidae	Tiger Puffer (*Takifugu rubripes*)	Kamijo et al., 2011	+
Obestatin	Cypriniforme	Cyprinidae	Grass carp (*Ctenopharyngodon idellus*)	Yuan et al., 2015	− ?
Octadecaneuropeptide (ODN)	Cypriniforme	Cyprinidae	Goldfish (*Carassius auratus*)	Matsuda et al., 2007b, 2010, 2011b	−
Orexin	Characiforme	Characidae	Blind cavefish (*Astyanax fasciatus mexicanus*)	Wall and Volkoff, 2013; Penney and Volkoff, 2014	+
			Dourado (*Salminus brasiliensis*)	Volkoff et al., 2016	+
		Serrasalmidae	Pacu (*Piaractus mesopotamicus*)	Volkoff et al., 2017	+
			Pirapitinga (*Piaractus brachypomus*)	Volkoff, 2015a	?
			Red-bellied piranha (*Pygocentrus nattereri*)	Pérez Sirkin et al., 2013; Suzuki and Yamamoto, 2013; Volkoff, 2014a	+
	Cypriniforme	Cyprinidae	Goldfish (*Carassius auratus*)	Abbott and Volkoff, 2011; Facciolo et al., 2011; Hoskins and Volkoff, 2012; Crudo et al., 2013; Volkoff, 2013, 2014b; Nisembaum et al., 2014; D'angelo et al., 2016	+
			Zebrafish (*Danio rerio*)	Panula, 2010; Yokobori et al., 2011; Elbaz et al., 2012; Pavlidis et al., 2015; Sterling et al., 2015	+
	Gadiforme	Gadidae	Cod (*Gadus morhua*)	Tuziak et al., 2014; Le et al., 2016	+
	Perciforme	Cichlidae	*Astatotilapia burtoni*	Grone et al., 2012	+
			*Cichlasoma dimerus*	Pérez Sirkin et al., 2013	?
			Nile tilapia (*Oreochromis niloticus*)	Chen et al., 2011	+
		Labridae	Cunner (*Tautogolabrus adspersus*)	Babichuk and Volkoff, 2013; Hayes and Volkoff, 2014	+
			Orange-spotted grouper (*Epinephelus coioides*)	Yan et al., 2011	+
			Ornate wrasse (*Thalassoma pavo*)	Facciolo et al., 2009	+
	Pleuronectiforme	Pleuronectidae	Winter flounder (*Pseudopleuronectes americanus*)	Buckley et al., 2010	+
			Barfin Flounder (*Verasper moseri*)	Amiya et al., 2012	+
	Polypteriformes actinopterygian	Polypteridae	*Polypterus senegalus* and *Erpetoichthys calabaricus*	López et al., 2014	?
	Rajiformes	Rajidae	Winter skate (*Leucoraja ocellata*)	MacDonald and Volkoff, 2010	+
	Salmoniforme	Salmonidae	Rainbow trout (*Oncorhynchus mykiss*)	Varricchio et al., 2015	?
PACAP	Cypriniforme	Cyprinidae	Goldfish (*Carasisus auratus*)	Matsuda et al., 2005; Matsuda and Maruyama, 2007	−
			Grass carp (*Ctenopharyngodon idellus*)	Zhou et al., 2013	− ?
	Gadiforme	Gadidae	Cod (*Gadus morhua*)	Xu and Volkoff, 2009	− ?
POMC/α-MSH	Cypriniforme	Cyprinidae	Goldfish (*Carasisus auratus*)	Cerdá-Reverter et al., 2003; Kang et al., 2010; Kojima et al., 2010; Yan et al., 2016	−
			Zebrafish (*Danio rerio*)	Shanshan et al., 2016	−
			Zebrafish (*Danio rerio*)	Dalmolin et al., 2015	+
	Pleuronectiforme	Pleuronectidae	Barfin flounder (*Verasper moseri*)	Takahashi et al., 2005	0
			Halibut (*Hippoglossus hippoglossus)*	Gomes et al., 2015	?
			Olive flounder (*Paralichthys olivaceus*)	Kang and Kim, 2015	0
	Salmoniforme	Salmonidae	Atlantic salmon (*Salmo salar*)	Valen et al., 2011	−
			Coho salmon (*Oncorhynchus kisutch*)	Kim et al., 2015	0
			Coho salmon (*Oncorhynchus kisutch*)	Leder and Silverstein, 2006; White et al., 2016	−
PrRP	Cypriniforme	Cyprinidae	Goldfish (*Carassius auratus*)	Kelly and Peter, 2006	−
	Perciforme	Gobidae	Mudskipper (*Periophtalmus modestus*)	Sakamoto et al., 2002; Tachibana and Sakamoto, 2014	+ ?
PYY	Acipenseriformes	Acipenseridae	Siberian sturgeon (*Acipenser baerii*)	Chen et al., 2015	−
	Characiforme	Characidae	Blind cavefish (*Astyanax fasciatus mexicanus*)	Wall and Volkoff, 2013	−
		Serrasalmidae	Red-bellied piranha (*Pygocentrus nattereri*)	Volkoff, 2014a	−
	Clupeiformes	Engraulidae	Tapertail anchovy (*Coilia nasus)*	Yang et al., 2016	+ ?
	Cypriniforme	Cyprinidae	Goldfish (*Carassius auratus*)	Gonzalez and Unniappan, 2010	−
			Grass carp (*Ctenopharyngodon idellus*)	Chen et al., 2013, 2014	−
	Perciforme	Carangidae	Yellowtail (*Seriola quinqueradiata*)	Murashita et al., 2006, 2007	+ ?
	Salmoniforme	Salmonidae	Atlantic salmon (*Salmo salar*)	Murashita et al., 2009a; Valen et al., 2011; Kousoulaki et al., 2013	0
	Siluriforme	Ictaluridae	Channel catfish (*Ictalurus punctatus*)	Schroeter et al., 2015	0 ?
Secretoneurin	Cypriniforme	Cyprinidae	Goldfish (*Carassius auratus*)	Trudeau et al., 2012; Mikwar et al., 2016	+
Spexin	Cypriniforme	Cyprinidae	Goldfish (*Carassius auratus*)	Wong et al., 2013	−
	Perciforme	Labridae	Orange-spotted grouper (*Epinephelus coioides*)	Li S. et al., 2016	−
Thyroid axis	Acipenseriformes	Acipenseridae	Amur sturgeon (*Acipenser schrenckii*)	Li et al., 2012	?
	Cypriniforme	Cyprinidae	Goldfish (*Carassius auratus*)	Sinha et al., 2012	+
			Goldfish (*Carassius auratus*)	Abbott and Volkoff, 2011; Goodyear, 2012	+ ?
	Siluriforme	Ictaluridae	Channel catfish (*Ictalurus punctatus*)	Pohlenz et al., 2013	+

The effects on feeding are described as stimulatory (+), inhibitory (−), not detected (0) or unknown/uncertain (?).

### Major appetite regulating factors

#### Central orexigenic factors

##### Agouti-related protein (or peptide, AgRP)

AgRP is a peptide released by hypothalamic NPY/AgRP neurons and is an endogenous antagonist of the melanocortin receptors MC3R and MC4R. AgRP plays a crucial role in the regulation of energy balance, as it increases food intake, by antagonizing the effects of the anorexigenic POMC product, α-melanocyte-stimulating hormone (α-MSH) (Sohn, 2015; Takeuchi, 2016).

In fish, AgRP has been identified in several species, including teleosts (e.g., goldfish *Carassius auratus* Cerdá-Reverter and Peter, 2003 and zebrafish *Danio rerio* Song et al., 2003, Atlantic salmon *Salmo salar* Murashita et al., 2009a, and seabass *Dicentrarchus labrax* Agulleiro et al., 2014, pufferfish *Takifugu rubripes* Klovins et al., 2004; Kurokawa et al., 2006), who have two genes products (AgRP1 and AgRP2; Cérda-Reverter et al., 2011) and Holocephali (Chimaeriforme, elephant fish *Callorhinchus milii* Västermark and Schioth, 2011).

AgRP appears to act as an orexigenic factor in Cypriniformes, as fasting increases hypothalamic *AgRP* expression in goldfish (Cerdá-Reverter and Peter, 2003), zebrafish (Song et al., 2003), and Ya fish *Schizothorax prenanti* (Wei et al., 2013). In addition, transgenic zebrafish overexpressing AgRP exhibit obesity, increased growth and adipocyte hypertrophy (Song and Cone, 2007). GH-transgenic common carp *Cyprinus carpio*, which display increased food intake, have higher hypothalamic AgRP1 mRNA expression levels than non-transgenic fish, further suggesting an orexigenic action (Zhong et al., 2013). However, this is contradicted by another study in carp showing that brain AgRP mRNA expression decreases after fasting and increases after re-feeding (Wan et al., 2012). In seabass (Perciforme), long-term fasting increases hypothalamic expression of AgRP1 but decreases that of AgRP2 (Agulleiro et al., 2014), suggesting an isoform-specific orexigenic action.

Within Salmoniformes, there is conflicting data with regards to the actions of AgRP. In Arctic charr *Salvelinus alpinus*, non-feeding fish have higher brain AgRP expression levels than feeding fish (Striberny et al., 2015) and transgenic coho salmon *Oncorhynchus kisutch*, which display increased feeding, have higher brain AgRP1 levels of mRNA than wild-type fish (Kim et al., 2015), suggesting an orexigenic role for AgRP. However, in Atlantic salmon, AgRP-1 brain mRNA levels decrease after fasting (Murashita et al., 2009a) and increase after feeding (Valen et al., 2011), rather pointing to an anorexigenic role.

##### Galanin

Galanin is a peptide expressed in both central nervous system and GIT, that regulates diverse physiological functions in mammals, including arousal/sleep, feeding, energy metabolism, and reproduction (Merchenthaler, 2010). Galanin and its receptors have been identified in a number of fish species (see review in Mensah et al., 2010). Central injections of galanin stimulate feeding in Cypriniformes (both goldfish de Pedro et al., 1995; Volkoff and Peter, 2001b, and tench, *Tinca tinca* Guijarro et al., 1999). In goldfish, brain galanin mRNA expression is not affected by fasting but increases post-prandially in unfed fish (Unniappan et al., 2004) and in zebrafish, fasting up-regulates brain mRNA expression of galanin receptors (Li et al., 2013). These data suggest that the galanin system is involved in the regulation of feeding in Cypriniformes, and perhaps other fish.

##### Melanin concentrating hormone (MCH)

Melanin concentrating hormone is a peptide originally isolated from the pituitary of chum salmon (*Oncorhynchus keta*) as a hormone involved in body color change (Kawauchi et al., 1983). MCH was later isolated in mammals and shown to stimulate feeding (Qu et al., 1996). In fish, the role of MCH as an appetite regulator is still unclear.

In Cypriniformes, early immunoreativity (ir) studies in goldfish showed the presence of MCH in neuron populations related to the regulation of feeding and of sleep and arousal (Huesa et al., 2005). In goldfish, central injections of MCH decrease feeding but have no effect on locomotor activity (Shimakura et al., 2006), anti-MCH serum treatments increase feeding (Matsuda et al., 2007a), and the number of certain hypothalamic neuronal cell bodies containing MCH-ir decreases in fasted fish (Matsuda et al., 2007a), altogether suggesting an anorexigenic role for MCH in this species. However, in Ya fish, MCH hypothalamic mRNA expression is higher in fasted compared to fed fish, suggesting an orexigenic role (Wang et al., 2016). Data on Gadiformes and Pleuronectiformes also seem to suggest an appetite-stimulating role for MCH: MCH brain mRNA levels increase during fasting in both Atlantic cod *Gadus morhua* (Tuziak and Volkoff, 2013a) and winter flounder *Pseudopleuronectes americanus* (Tuziak and Volkoff, 2012), and in cod fed diets with relatively high amounts of plant (camelina) material (Tuziak et al., 2014). In starry (*Platichthys stellatus*; Kang and Kim, 2013b), olive (*Paralichthys olivaceus*; Kang and Kim, 2013a) and Barfin (*Verasper moseri;* Takahashi et al., 2004) flounders, fish placed in light backgrounds have enhanced appetite and growth, which is concomitant with increased expression levels of MCH mRNA and/or numbers of MCH neurons in the brain. However, in medaka *Oryzias latipes*, transgenic fish overexpressing MCH have normal growth and feeding behavior (Qu et al., 1996) and in the scalloped hammerhead shark *Sphyrna lewini*, hypothalamic MCH mRNA levels are not affected by fasting (Mizusawa et al., 2012), suggesting little or no role of MCH in feeding regulation of Beloniformes and sharks.

Neuronal relationship between MCH- and NPY-containing neurons have been shown in goldfish (Matsuda et al., 2009) and MCH treatment increases orexin mRNA expression and decreases NPY mRNA expression in cultured goldfish forebrain slices (Matsuda et al., 2009), suggesting an interaction of MCH with appetite regulators in goldfish. Similarly, in red-bellied piranha *Pygocentrus nattereri*, orexin and MCH co-localize in pituitary and brain (Suzuki et al., 2007), and in Barfin flounder, close contacts are seen between orexin- and MCH-ir cell bodies and fibers in the hypothalamus, suggesting an interaction between the two systems and a possible role for MCH in the modulation of locomotion and feeding (Amiya et al., 2008).

##### Neuropeptide Y (NPY)

Neuropeptide Y (NPY) belongs to the NPY family of peptides, which also includes, peptide YY and pancreatic polypeptide (PP) (Holzer et al., 2012). Originally isolated from mammalian brain extracts (Tatemoto et al., 1982), NPY is one of the most abundant neuropeptides within the brain and has a major regulatory role in energy homeostasis and food intake (Loh et al., 2015).

Although reports for NPY-like ir in fish brain and other tissues appear in the 1980's (e.g., Osborne et al., 1985; Danger et al., 1990), the first fish NPY cDNAs were reported in goldfish and the electric ray *Torpedo marmorata* (elasmobranch, Torpediniformes; Blomqvist et al., 1992). One of the first studies showing the role of NPY in regulating in fish was that of Silverstein et al., showing by *in situ* hybridization (ISH) that, in chinook salmon (*Oncorhynchus tshawytscha*) and coho salmon, NPY-like mRNA signal areas were greater in fasted than fed fish (Silverstein et al., 1998). The first *in vivo* injection studies were performed in goldfish (Lopez-Patino et al., 1999; de Pedro et al., 2000; Narnaware et al., 2000) and channel catfish *Ictalurus punctatus* (Silverstein and Plysetskaya, 2000). Since then, NPY has been one of the most studied appetite-regulating hormones in fish. It has been cloned and/or shown to regulate feeding in several groups, including Characiformes (Pereira et al., 2015), Cypriniformes [(e.g., goldfish, zebrafish (Yokobori et al., 2012), blunt snout bream *Megalobrama amblycephala* (Xu et al., 2016), grass carp *Ctenopharyngodon idellus* (Jin et al., 2015), Jian carp (*Cyprinus carpio*) (Tang et al., 2014), Ya fish (Wei et al., 2014)], Gadiformes (Atlantic cod Kortner et al., 2011; Tuziak et al., 2014); Gonorynchiformes (milkfish *Chanos chanos*, Lin et al., 2016); Perciformes (yellowtail *Seriola quinqueradiata* Hosomi et al., 2014, *Astatotilapia burtoni* Grone et al., 2012, cunner *Tautogolabrus adspersus* Babichuk and Volkoff, 2013, orange-spotted grouper *Epinephelus coioides* Tang et al., 2013, sea bass Leal et al., 2013, mandarin fish, *Siniperca chuatsi* Sun et al., 2014, cobia *Rachycentron canadum* Van Nguyen et al., 2013, gourami *Trichogaster pectoralis* Boonanuntanasarn et al., 2012); Pleuronectiformes (olive flounder Wang et al., 2015, winter flounder MacDonald and Volkoff, 2009a, Brazilian flounder *Paralichthys orbignyanus* Campos et al., 2012), Salmoniformes (e.g., rainbow trout *Oncorhynchus mykiss* Aldegunde and Mancebo, 2006, Atlantic salmon Valen et al., 2011; Kim et al., 2015), Siluriformes (channel catfish, Peterson et al., 2012; Schroeter et al., 2015); Tetraodontiformes (tiger puffer *Takifugu rubripes* Kamijo et al., 2011) as well as elasmobranchs [(e.g., winter skate *Leucoraja ocellata*, Rajiforme (MacDonald and Volkoff, 2009b) and spotted catshark (*Scyliorhinus canicula*, Carcharhiniforme) Mulley et al., 2014)] and holocephalans (elephant fish Chimaeriformes; Larsson et al., 2009). The majority of these studies indicate that NPY has a widespread distribution and is present in both brain and intestinal tract, that it acts as an orexigenic factor and that its expression is affected by feeding and fasting.

##### Orexin

Orexins (also called hypocretins) are neuropeptides originally isolated in rats (Sakurai, 2014), that have since been identified in several fish species. The first direct evidence of an orexigenic action of orexins was shown via intracerebroventricular (ICV) injections in goldfish (Volkoff et al., 1999). As in mammals (Tsujino and Sakurai, 2009; Sakurai, 2014), orexins increase not only appetite and feeding behavior but also locomotor activity and reward-seeking/foraging behavior in fish (Panula, 2010).

In both goldfish (Volkoff et al., 1999; Nakamachi et al., 2006; Facciolo et al., 2011) and zebrafish (*Danio rerio*) (Yokobori et al., 2011) (Cypriniformes), and cavefish (*Astyanax mexicanus*) (Characiforme) (Penney and Volkoff, 2014), orexin injections increase searching/feeding behaviors. In orange-spotted grouper (Perciforme), intraperitoneal (IP) orexin injections increase hypothalamic mRNA expression levels of NPY, a major appetite stimulator (Yan et al., 2011), further suggesting an orexigenic role. However, in ornate wrasse (*Thalassoma pavo)* (Perciforme), orexin IP injections induce increases in locomotion but decreases in feeding (Facciolo et al., 2009), suggesting that the major role of orexin might be induction of hyperactivity rather than increasing food ingestion. Indeed, in goldfish, hypothalamic orexin mRNA expression levels peak when fish are active prior to a scheduled meal (Hoskins and Volkoff, 2012) and in zebrafish, increased locomotor activity is associated with increased activity of hypothalamic orexin neurons (Naumann et al., 2010) and larvae overexpressing orexin are hyperactive (Woods et al., 2014). Similarly, orexin expression decreases post-feeding in Characiformes [cavefish (Wall and Volkoff, 2013), dourado (*Salminus brasiliensis*) (Volkoff et al., 2016) and pacu (*Piaractus mesopotamicus*) (Volkoff et al., 2017)] and is higher at mealtime in orange-spotted grouper (Yan et al., 2011) and tilapia (Chen et al., 2011) (Perciformes), as well as Atlantic cod (Gadiforme) (Xu and Volkoff, 2007). In cod, orexin levels are also higher during daylight hours, when animals are active (Hoskins and Volkoff, 2012).

Fasting increases orexin brain mRNA expression in Cypriniformes (goldfish Abbott and Volkoff, 2011 and zebrafish Yokobori et al., 2011), Characiformes (cavefish Wall and Volkoff, 2013, dourado Volkoff et al., 2016, pacu Volkoff et al., 2017, and red-bellied piranha Volkoff, 2014a), and Pleuronectiformes (winter flounder Buckley et al., 2010 and Barfin flounder Amiya et al., 2012). In the mouth-brooding *Astatotilapia burtoni* (Perciforme), brain orexin mRNA levels increase in non-feeding females carrying eggs (Grone et al., 2012). In Atlantic cod (Gadiforme), orexin brain expression levels are higher in fish fed low rations than in fish fed high rations (Xu and Volkoff, 2007) or in fish fed the 30% camelina (plant) meal diet compared to fish fed a control (fish) diet (Tuziak et al., 2014), suggesting an effect of food quality and quantity on orexin expression. However, torpid cunner (Peciforme, labridae) undergoing a long-term fasting have low brain and gut orexin expression levels (Babichuk and Volkoff, 2013; Hayes and Volkoff, 2014), but this decrease might be due to a toprpor-induced general metabolic shutdown.

Anatomical studies provide further evidence for a role of orexin in nutrient digestion/abrorption and growth. In several fish species, e.g., pirapitinga (*Piaractus brachypomus*) (Characiforme) (Volkoff, 2015a), cunner (Perciforme) (Hayes and Volkoff, 2014) and rainbow trout (Salmoniforme) (Varricchio et al., 2015), orexin mRNA/protein expression is high in the gastrointestinal tract, suggesting a role of the orexin system in regulating feeding and digestive processes. Among Perciformes, in Japanese sea perch (*Lateolabrax japonicus*), orexin-like ir is present in pituitary GH-containing cells, suggesting a control of growth by the orexin system (Suzuki et al., 2007) and in *Cichlasoma dimerus*, orexin-ir fibers are present in both hypothalamus and in pituitary, suggesting a neuroendocrine control of pituitary secretions (Pérez Sirkin et al., 2013).

In addition to teleosts, orexin has been examined in the primitive bony fish birchir *Polypterus senegalus* and rope fish *Erpetoichthys calabaricus* (Chondrosteans, Polypteriformes) for which the brain orexin ir patterns are similar to that of other fish examined (López et al., 2014) and in the Chondrichthyan winter skate (Rajiforme), in which fasting increases hypothalamic orexin expression (MacDonald and Volkoff, 2010).

Overall, it appears that in all fish species studied to date, orexin is related to both food intake and appetitive/searching behavior and perhaps to growth.

#### Anorexigenic factors

##### CART

CART is a peptide which transcript expression is regulated by administration of cocaine or amphetamine in rodents (Vicentic and Jones, 2007; Subhedar et al., 2014) and amphetamine in goldfish (Volkoff, 2013). CART acts as an anorexigenic factor in mammals (Larsen and Hunter, 2006), and was first identified and shown to be anorexigenic in goldfish (Volkoff and Peter, 2000, 2001a).

Two CART isoforms have been identified in goldfish (Volkoff and Peter, 2001a) and common carp (Wan et al., 2012), and 4 in zebrafish (Akash et al., 2014) whereas, to date, only one form has been isolated for grass carp (Zhou et al., 2013; Liu et al., 2014), Characiformes [pirapitinga (serrasalmidae) (Volkoff, 2015a), pacu (serrasasalmidae) (Volkoff et al., 2017) and dourado (characidae) (Volkoff et al., 2016), red bellied piranha (serrasalmidae) (Volkoff, 2014a)], Salmoniformes [Atlantic salmon (Murashita et al., 2009a), rainbow trout (Figueiredo-Silva et al., 2012), Arctic charr (Striberny et al., 2015) and lake trout (*Salvelinus namaycush*) (Volkoff et al., 2007)], Siluriformes (channel catfish Kobayashi et al., 2008), Gadiformes (Atlantic cod Kehoe and Volkoff, 2007), Perciformes (cunner Babichuk and Volkoff, 2013), winter flounder (MacDonald and Volkoff, 2009a) and Atlantic halibut (*Hippoglossus hippoglossus*) (Gomes et al., 2015) (Pleuronectiformes), venomous toadfish *Thalassophryne nattereri* (Batrachoidiforme) (Magalhaes et al., 2006), rainbow smelt (*Osmerus mordax*) (Osmeriforme), pufferfishes (*Takifugu rubripes* and *Tetraodon nigroviridis*, Tetraodontiforme) and stickleback *Gasterosteus aculeatus* (Gasterosteiforme) (cited in Murashita et al., 2009a). However, six forms of CART have been identified in the medaka (Beloniforme) (Murashita and Kurokawa, 2011) and seven forms in Senegalese sole *Solea senegalensis* (Pleuronectiforme), the highest number of CART genes reported to date in a vertebrate species (Bonacic et al., 2015). The only elasmobranch CART identified to date is that of winter skate (Rajiforme) (MacDonald and Volkoff, 2009b).

CART injections induce a decrease in food intake and an increase in locomotion in goldfish (Volkoff and Peter, 2000) and enhance responsiveness to sensory stimuli in zebrafish larvae (Woods et al., 2014), suggesting that CART is involved in feeding/searching behaviors in cyprinids.

Fasting/food restriction decreases CART brain expression in Cypriniformes (goldfish Volkoff and Peter, 2001a, zebrafish Nishio et al., 2012; Guillot et al., 2016 and common carp, Wan et al., 2012), most Characiformes (red-bellied piranha Volkoff, 2014a, and pacu Volkoff et al., 2017), most Salmoniformes (Atlantic salmon, Murashita et al., 2009a; Kousoulaki et al., 2013, rainbow trout Figueiredo-Silva et al., 2012), Atlantic cod (Kehoe and Volkoff, 2007), cunner (Perciforme) (Babichuk and Volkoff, 2013), medaka (CART3) (Murashita and Kurokawa, 2011), and Siluriformes (channel catfish Kobayashi et al., 2008, African sharptooth catfish *Clarias gariepinus* Subhedar et al., 2011), suggesting an anorexigenic role for CART in teleost fish. Postprandial increases in CART brain expression have been shown in Senegalese sole (CART1a, CART 2a and CART4) (Bonacic et al., 2015), pacu (Volkoff et al., 2017), dourado (Volkoff et al., 2016), channel catfish (Peterson et al., 2012) but not in cod (Kehoe and Volkoff, 2007).

However, in Arctic charr, CART hypothalamic expression is similar throughout the seasonal feeding cycles (Striberny et al., 2015) and fasting does not affect CART expression in either dourado (Volkoff et al., 2016), winter flounder (MacDonald and Volkoff, 2009a) or Atlantic halibut larvae (Gomes et al., 2015), and in lake trout, fish exposed to the pesticide tebufenozide and control fish have similar food intakes, despite higher CART mRNA brain expression levels in exposed fish (Volkoff et al., 2007). In winter skate, 2 weeks of fasting have no effects on brain CART expression (MacDonald and Volkoff, 2009b), suggesting that CART might not have a major feeding-regulating role in elasmobranchs.

CART expression does not appear to be affected by diet, as in both cod fed a camelina (plant) diet (Tuziak et al., 2014) or rotifers or zooplankton (Katan et al., 2016) and pacu fed soybean concentrate (Volkoff et al., 2017), similar CART brain expression are seen between experimental and control fish.

Overall, there is a large interspecific variation in the number of forms and responses to fasting in the CART system in fish, although most studies tend to show that CART is mostly a central factor that might act as an appetite inhibitor.

##### Pro-opiomelanocortin (POMC) family of peptides

Proopiomelanocortin (POMC) is a common precursor that is processed post-translationally to generate melanocortin peptides [α-, β-, and γ-melanocyte-stimulating hormone (α-, β-, γ-MSH)], adrenocorticotropic hormone (ACTH) and other hormones that include β-endorphin (β-END) and β-lipotropic hormone (β-LPH) (Adan et al., 2006; Takahashi, 2016). POMC is mainly produced in the vertebrate pituitary, but is also found in brain, in particular the arcuate nucleus (ARC) of the hypothalamus. Receptors for melanocortin peptides include five subtypes (MC1R- MC5R) (Takahashi, 2016). In mammals, POMC and α-MSH have been shown to be involved in the regulation of appetite and energy homeostasis: POMC neurons suppress appetite by releasing α-MSH, which is an agonist at the anorectic melanocortin-4 receptor (MC4R) (Adan et al., 2006; Cone, 2006; Sohn, 2015).

Teleost fish lack γ-MSH and the POMC gene encodes an extra MSH (δ-MSH) in elasmobranchs (Cérda-Reverter et al., 2011). Fish POMC was first identified in Salmoniformes (Kawauchi, 1983; Kitahara et al., 1988) and Cypriniformes (Arends et al., 1998), followed by the identification of several forms in other fish species. As in other vertebrates, fish POMC is mainly expressed in the pituitary gland, but also within the lateral tuberal nucleus, which is equivalent to the mammalian ARC (Cérda-Reverter et al., 2011). POMC, α-MSH and the MC4R have been shown to regulate feeding in a few fish species.

In goldfish, fasting does not seem to affect hypothalamic POMC mRNA expression levels (Cerdá-Reverter et al., 2003), but ICV administration of [Nle4, d-Phe7]- α-MSH, a melanocortin agonist, inhibits food intake (Cerdá-Reverter et al., 2003), suggesting the melanocortin system participates in central regulation of food intake in Cypriniformes (Cerdá-Reverter et al., 2003). In addition, ICV injections of a MSH (MC4R) receptor agonist (melanotan II) suppress hypothalamic NPY expression (Kojima et al., 2010), and hypothalamic α-MSH-containing neurons are in close contact to NPY-containing nerve fibers, suggesting that the anorexigenic actions of the melanocortin system are mediated in part by an inhibition of the NPY system. In zebrafish larvae, although early ISH studies could not detect fasting-induced changes in hypothalamic POMC transcript levels (Song et al., 2003), more recent qPCR studies indicate that POMCa expression decreases in starved fish (Shanshan et al., 2016). In addition, GH-transgenic zebrafish, who have increased feeding, display down-regulation of POMC (Dalmolin et al., 2015), consistent with an anorexigenic role for POMC-derived peptides in Cypriniformes.

Similarly, in salmonids, POMC/α-MSH appears to have an anorexigenic role. In coho salmon, IP injections of α-MSH decrease food intake (White et al., 2016), in rainbow trout, fasting induces a decrease in hypothalamic expression of POMC-A1 (but not POMC-A2 or POMC-B) (Leder and Silverstein, 2006), and in Atlantic salmon, expression of both POMC-A1 and POMC-B increase after feeding (Valen et al., 2011). Interestingly, α-MSH treatment does not affect feeding of GH-transgenic coho salmon (White et al., 2016), despite similar hypothalamic POMC and MC4R mRNA expression levels compared to non-transgenic fish (Kim et al., 2015), suggesting that the actions of α-MSH might be inhibited by high expression levels of GH and/or AgRP.

In both olive (Kang and Kim, 2015) and Barfin flounder (Takahashi et al., 2005) (Pleuronectiformes), pituitary POMC-C (isoforms 1, 2, and 3) mRNAs are not affected by fasting, suggesting pituitary POMC might not directly related to appetite regulation. However, in fasted halibut larvae, whole brain POMC-C mRNA expression is higher in unfed fish 30 min after re-feeding compared to continuously fed fish (Gomes et al., 2015), suggesting a short-term regulation of appetite. Given the small number of studies available, and the variation in experimental protocols (adults vs. larvae, pituitary vs. brain, long-term vs. short-term feeding), conclusions are difficult to drawn regarding the role of POMC in flatfish.

### Major peripheral factors

#### Ghrelin

Originally discovered in rat stomach as an endogenous ligand to the GH secretagogue-receptor (Kojima et al., 1999) ghrelin is the only known orexigenic factor in the GIT of mammals (Higgins et al., 2007). In the 2000's, a ghrelin-like peptide which stimulated GH release was first described in Nile tilapia (*Oreochromis mossambicus*; Shepherd et al., 2000) and a ghrelin-ir peptide was first detected in burbot (*Lota lota*) plasma (Mustonen et al., 2002). Using goldfish as a model, Unniappan et al. provided the first fish ghrelin cDNA sequence and the first evidence of an orexigenic role for ghrelin in fish, as central injections of ghrelin stimulated food intake (Unniappan et al., 2002). Subsequent studies on several fish species reported sequences for ghrelin and confirmed its role as an appetite stimulator in fish (see Jönsson, 2013 for a review), including other Cypriniformes [e.g., goldfish (Kang et al., 2011; Nisembaum et al., 2014; Blanco et al., 2016a); gibel carp (*Carassius auratus gibelio*) (Zhou et al., 2016); *Schizothorax davidi* (Zhou et al., 2014)], Characiformes (red-bellied piranha Volkoff, 2015b), Perciformes (Nile tilapia Schwandt et al., 2010), for which fasting-induced and periprandial changes in expression/protein levels occur. In Salmoniformes, there is contradictory evidence. In rainbow trout, central ghrelin injections and long-term peripheral treatment both decrease food intake compared to controls (Jönsson et al., 2010) and in Atlantic salmon, ghrelin plasma levels are lower in fasted fish compared with fed fish (Hevrøy et al., 2011) and show no clear periprandial changes (Vikesa et al., 2015), suggesting that ghrelin might have little effect or an inhibitory effect on feeding of in salmonids. In contrast, in brown trout (*Salmo truta*), ghrelin treatment increases foraging activity (Tinoco et al., 2014a). In rainbow trout, ICV ghrelin injections induce changes in parameters related to hepatic lipid metabolism (Velasco et al., 2016), suggesting a role of ghrelin in metabolism and nutrient storage. In yellow catfish (*Pelteobagrus fulvidraco*) (Siluriforme), although fasting increases ghrelin expression (Zhang et al., 2016a), no periprandial differences in plasma or stomach ghrelin expression are observed (Peterson et al., 2012).

It thus seems that the role of ghrelin in the regulation of feeding and metabolism of fish is still unclear, and might be species- and form-specific, so that further studies on more species are required.

#### Anorexigenic factors

##### Cholecystokinin (CCK)

In mammals, CCK inhibits food intake and induces the release of digestive enzymes from intestine/pancreas and gallbladder (Boguszewski et al., 2010; Dockray, 2012).

In fish, CCK was first shown to have a role in digestion, as, for example, it stimulated contraction of the gallbadder in coho (Vigna and Gorbman, 1977) and Atlantic (Aldman and Holmgren, 1987) salmon, as well as bluegill (*Lepomis macrochirus*), killifish (*Fundulus heteroclitus*), and the holostean bowfin (*Amia calva*) (Rajjo et al., 1988), stimulated lipase secretion in the stomachless killifish (Honkanen et al., 1988) and inhibited gastric secretion in Atlantic cod (Holstein, 1982). The first direct evidence of the actions of CCK on feeding was provided by injections in goldfish (Himick and Peter, 1994), followed by cloning of goldfish CCK cDNA (Peyon et al., 1998) and the demonstration of periprandial variations in CCK mRNA expression levels (Peyon et al., 1999). Subsequently, a number of studies have characterized CCK in several fish, including other Cypriniformes (e.g., common carp Zhong et al., 2013; zebrafish Koven and Schulte, 2012; Tian et al., 2015; grass carp; blunt snout bream Ping et al., 2013; Ji et al., 2015), Characiformes (e.g., cavefish Wall and Volkoff, 2013, dourado Pereira et al., 2015; Volkoff et al., 2016, thin dogfish *Oligosarcus hepsetus* Vieira-Lopes et al., 2013, pirapitinga Volkoff, 2015a, red-bellied piranha Volkoff, 2014a, pacu Volkoff et al., 2017), Salmoniformes (e.g., Atlantic salmon Valen et al., 2011), Gadiformes (Atlantic cod Tillner et al., 2013), Perciformes [e.g., yellowtail (Furutani et al., 2013; Hosomi et al., 2014); *Astatotilapia burtoni* (Grone et al., 2012); cunner (Babichuk and Volkoff, 2013; Hayes and Volkoff, 2014); sea bass (Tillner et al., 2014); yellow croaker (*Larimichthys crocea*) (Cai et al., 2015); white sea bream, *Diplodus sargus* (Micale et al., 2012, 2014)], Pleuronectiformes (e.g., winter flounder (MacDonald and Volkoff, 2009a), Atlantic halibut Kamisaka et al., 2001, olive flounder Kurokawa et al., 2000) and Siluriformes (channel catfish Peterson et al., 2012).

Overall, in all fish species studied to date, CCK appears to have similar roles in feeding and digestive processes to its role in mammals, i.e., it acts as a satiety/appetite-inhibiting factor and induces the release of digestive enzymes from the GIT.

##### Leptin

Leptin, a peptide originally cloned in obese *ob/ob* mice (Zhang et al., 1994), is secreted in mammals mainly by white adipose tissue, and its blood levels are proportional to body fat content (Park and Ahima, 2015). Leptin is a multifunctional hormone in both mammals (Park and Ahima, 2015) and fish (see review by Gorissen and Flik, 2014) and is involved in the regulation of not only food intake and body weight, but also reproduction, development and stress responses.

First hints of a role of leptin in fish were provided by reports of a decrease in feeding in goldfish ICV-injected with human leptin (Volkoff et al., 2003). The first fish leptin was identified in the pufferfish genome in 2005 by synteny studies (Kurokawa et al., 2005), followed by isolation of zebrafish, medaka, and carp leptins (Huising et al., 2006b). Since then, leptins have been identified in several fish species and shown to have multiple physiological functions (reviewed in Copeland et al., 2011; Angotzi et al., 2013; Londraville et al., 2014). As opposed to mammals who have a single leptin gene, several fish species have several leptin gene paralogs (e.g., lepA and lepB). Also in contrast to mammals, where subcutaneous fat is the main source of leptin, fish leptin is expressed in several tissues including liver and intestine, which is consistent with the fact that fish generally store lipids in intra-abdominal regions and liver (Birsoy et al., 2013).

Most studies on fish leptin have been conducted in Cypriniformes, in particular goldfish and zebrafish, and Salmoniformes. In goldfish, leptin injections decrease feeding and locomotor behavior (Volkoff et al., 2003; de Pedro et al., 2006; Vivas et al., 2011; Tinoco et al., 2012) in part by stimulating anorexigenic sytems (e.g., CART, CCK, and POMC) and inhibiting orexigenic ones (e.g., orexin, NPY, AgRP) (Volkoff et al., 2003; Yan et al., 2016). Similarly, in rainbow trout (Salmoniforme), central leptin administration suppresses food intake and increases the hypothalamic expressions of CART and POMC (Gong et al., 2016). Leptin treatment also inhibits feeding in grass carp (Li et al., 2010) (Cypriniforme) and increases energy expenditure in zebrafish larvae (Renquist et al., 2013). In Atlantic salmon (Salmoniforme), chronic IP treatment with leptin induces a decrease in growth rates (Murashita et al., 2011), and in hybrid striped bass (*Morone saxatilis* × *Morone chrysops*) (Perciforme), leptin treatment increases hepatic IGF-1 mRNA expression (Won et al., 2016), suggesting that leptin affects metabolism and growth.

Hepatic/gut/brain leptin increases in expressions are seen post-prandially in goldfish (Tinoco et al., 2012, 2014b), common carp (Huising et al., 2006a) and zebrafish (Tian et al., 2015) (Cypriniformes) as well as pacu (Volkoff et al., 2017) (Characiforme). However, in rainbow trout plasma leptin levels decrease post-feeding (Johansson and Björnsson, 2015).

There is a great variability in results with regards to fasting-induced changes in the leptin system. In goldfish, no significant differences in either brain or liver leptin expressions are seen between control, overfed and fasting fish, suggesting nutritional status does not affect the leptin system in goldfish (Tinoco et al., 2012). Similarly, leptin expression is not affected by fasting in the liver of common carp (Huising et al., 2006a) (Cyrpiniforme) and Nile tilapia (Shpilman et al., 2014) (Perciforme) or in the brains of red-bellied piranha (Volkoff, 2015b) and pacu (Volkoff et al., 2017) (Characiformes). However, fasting/food restriction increases hepatic leptin expression in white-clouds mountain minnow (*Tanichthys albonubes*, Cypriniforme; Chen et al., 2016b), in most Perciformes examined (orange-spotted grouper Zhang et al., 2013, mandarin fish Yuan et al., 2016, and mackerel *Scomber japonicus* Ohga et al., 2015, European sea bass Gambardella et al., 2012), in Arctic charr (Jørgensen et al., 2013) and Atlantic salmon (Rønnestad et al., 2010; Trombley et al., 2012; Moen and Finn, 2013) (Salmoniformes). In contrast, decreases in leptin expression are seen in liver of zebrafish (lepA) (Gorissen et al., 2009) and striped bass (*Morone saxatilis*) (lepB, perciforme) (Won et al., 2012) and intestine of red-bellied piranha (Volkoff, 2015b), and in blunt snout bream (Cypriniforme), higher feeding rates are associated with increased leptin pituitary expression (Xu et al., 2016). Whereas plasma leptin levels increase following fasting in rainbow trout (Salmeron et al., 2015; Johansson et al., 2016; Pfundt et al., 2016), Atlantic salmon (Trombley et al., 2012) and fine flounder *Paralichthys adspersus* (Pleuronectiforme) (Fuentes et al., 2012, 2013), they have been shown to decrease in earlier studies in fasted burbot (*Lota lota*) (Gadiforme) (Nieminen et al., 2003) and green sunfish (*Lepomis cyanellus)* (Perciforme) (Johnson et al., 2000).

In fish, leptin has been linked to metabolism. For example, in zebrafish, knocking down lepA decreases metabolic rate (Dalman et al., 2013) and in golden pompano, *Trachinotus blochii* (Perciforme), lepA gene polymorphisms are associated with different body weights, heights and lengths (Wu et al., 2016). Whereas in mammals, leptin acts as an adipostat and its plasma levels are proportional to the amount of body fat, there is little evidence for such a role in fish. In topmouth culter *Culter alburnus* (Cyprinoforme), leptin mRNA expression is lower in wild populations, who have more muscle fat content than cultured fish (Wang et al., 2013), in grass carp, fish fed high fat diets have higher leptin expression (Li A. et al., 2016) than control fish, and in medaka, leptin receptor null-mutants have higher food intake and larger deposits of visceral fat than that of wild-type fish (Chisada et al., 2014), suggesting a correlation between leptin levels and fat. However, results from other studies seem to contradict this hypothesis: leptin receptor null adult zebrafish do not exhibit increased feeding or adiposity (Michel et al., 2016); In rainbow trout, leptin levels are higher in lean fish than fat fish (Salmeron et al., 2015; Johansson et al., 2016; Pfundt et al., 2016), and in Arctic charr, neither hepatic leptin expression nor plasma leptin levels correlate with fish adiposity (Froiland et al., 2012; Jørgensen et al., 2013); In murray cod *Maccullochella peelii peelii* (Perciforme), fish fed different experimental diets containing fish oil with or without vegetable oil have similar leptin levels (Ettore et al., 2012; Varricchio et al., 2012); In yellow catfish (Siluriforme), IP injections of human leptin reduce hepatic lipid content and the activities of lipogenic enzymes (Song et al., 2015) but Zn deficiency, which tends to increase hepatic and muscle lipid contents, does not affect leptin mRNA levels (Zheng et al., 2015).

Zebrafish lacking a functional leptin receptor have alterations in insulin and glucose levels, suggesting a role of leptin in the control of glucose homeostasis (Michel et al., 2016), which is consistent with data showing that leptin gene expression is induced by glucose in grass carp (Lu et al., 2015) and that leptin injections increase plasma glucose levels in Nile tilapia (Baltzegar et al., 2014).

Interestingly, in the Gymnotiforme *Eigenmannia virescens*, intramuscular injections of leptin increase electric organ discharges (EOD) amplitude in food-deprived but not well-fed fish, suggesting that leptin mediates EOD responses to metabolic stress in electric fish (Sinnett and Markham, 2015).

Overall, there seems to be a great species-specific variability in the functions of leptin with regards to the regulation of feeding and metabolism in fish, perhaps due to different lipid metabolism and storage areas among fish species.

##### Peptide YY

Peptide YY consists of two forms, PYYa and PYYb (previously called PY) (Wahlestedt and Reis, 1993; Cerdá-Reverter and Larhammar, 2000; Sundström et al., 2013) and is a brain-gut peptide that acts as an anorexigenic signal in mammals (Blevins et al., 2008; Karra et al., 2009; Zhang et al., 2012). Interestingly, one of the first studies showing an effect of PYY on feeding in mammals used fish PYY (Balasubramaniam et al., 1992). PYY was first shown to be present in the gastrointestinal tract of fish by immunochemical methods in the 1980's (daddy sculpin *Cottus scorpius* and Baltic sea cod *Gadus morhua callarias* El-Salhy, 1984) and first cloned and detected in the brain by ISH in an Agnatha, the river lamprey (*Lampetra fluviatilis*; Söderberg et al., 1994). The first indirect evidence of a role for PYY in feeding in fish was provided in sea bass, in which PYY transcripts were detected in brain areas regulating feeding (Cerdá-Reverter et al., 2000) and the first direct evidence of an anorexigenic role for PYY in fish was provided by IP injections of goldfish PYY in goldfish (Gonzalez and Unniappan, 2010). Peripheral injections of species-specific PYY also decrease food intake in another cyprinid, the grass carp (Chen et al., 2013) and in Siberian sturgeon *Acipenser baerii* (Acipenseriformes) (Chen et al., 2015). However, in channel catfish (Siluriformes), human PYY injections do not affect food intake or plasma glucose levels or hypothalamic POMC expression (Schroeter et al., 2015), suggesting perhaps that species-specific PYYs are needed to elicit an effect on feeding.

Fasting induces decreases in brain PYY expression in both goldfish (Gonzalez and Unniappan, 2010) and Ya fish (Yuan et al., 2014) (Cypriniformes) and in PPY intestinal expression in red-bellied piranha (Characiforme,) (Volkoff, 2014a), suggesting a role in satiety. However, fasting does not affect brain PYY expression in either cavefish (Characiforme) (Wall and Volkoff, 2013) or red-bellied piranha (Volkoff, 2014a), either brain or gut PYY mRNA expression in Atlantic salmon (Salmoniforme) (Murashita et al., 2009b), and induces increases in PYY gut expression in both yellowtail (Perciformes) (Murashita et al., 2006, 2007) and Japanese grenadier anchovy *Coilia nasus* (Clupeiformes) (Yang et al., 2016).

PYY mRNA expression increases post-feeding in the brain of goldfish (Gonzalez and Unniappan, 2010) and Ya fish (Yuan et al., 2014), cave fish (Wall and Volkoff, 2013) and Siberian stur*geon* (Chen et al., 2015), in the intestine of grass carp (Chen et al., 2014) and in whole larval Atlantic halibut (Pleuronectiformes) (Gomes et al., 2015). However, in Atlantic salmon, brain PYY expression shows no periprandial changes (Valen et al., 2011; Kousoulaki et al., 2013), perhaps suggesting that PYY does not play a major role as a short-term satiety factor in salmonids.

Overall, it appears that in most fish examined to date, PYY might acts as an anorectic/satiety peptide, although this does not seem to hold true for all fish species (e.g., salmon, yellowtail, or catfish).

### Other hormones and systems

#### Hypothalamus-pituitary-thyroid axis (HPT axis)

The hypothalamic-pituitary-thyroid (HPT) axis regulates levels of thyroid hormones, which are essential for a number of biological functions, including food intake and energy expenditure. Hormones produced by the axis consist of thyrotropin releasing hormone (TRH), thyroid stimulating hormone (TSH) and thyroid hormones (triiodothyronine T_3_ and thyroxine T_4_) secreted by the hypothalamus, the pituitary and the thyroid gland, respectively (Fekete and Lechan, 2014).

In goldfish (Cypriniforme), ICV injections of TRH increase feeding and locomotor behaviors and the hypothalamic mRNA expressions of both orexin and CART (Abbott and Volkoff, 2011), and IP injections of T_4_ increase food intake and locomotion (Goodyear, 2012), suggesting an orexigenic role. Fasting increases TRH hypothalamic mRNA levels (Abbott and Volkoff, 2011), further suggesting that the HPT axis regulates feeding in goldfish. In Amur sturgeon, *Acipenser schrenckii* (Acipenseriforme), lower serum levels of thyroid hormones are seen in fish placed in high-density groups who display low feeding rates (Li et al., 2012). However, decreases in plasma levels of thyroid hormones are seen in fasted goldfish [T_3_] (Sinha et al., 2012) and in fasted channel catfish [T_4_ and T_3_] (Gaylord et al., 2001), suggesting that food deprivation might decrease the activity of the HPT at the level of thyroid hormone synthesis and secretion, similar to what is observed in mammals (Boelen et al., 2008). A decrease in circulating thyroid hormones might inhibit the thyroid hormone negative feedback action on hypothalamic cells and contribute to the increase in hypothalamic TRH expression levels seen in goldfish. Overall, these data suggest that, in fish, TRH and thyroid hormones might affect feeding and metabolism and that nutritional status might affect the HPT axis.

#### Reproductive hypothalamus-pituitary-gonad (HPG) axis

##### Gonadotropin releasing hormone (GnRH)

GnRH is a hypothalamic hormone that stimulates the release of pituitary gonadotropins, which in turn stimulate the release of gonadal steroids. Three major forms of GnRH are present in fish, GnRH 1, 2, and 3 (Roch et al., 2014). GnRH appears to act as an anorexigenic hormone, as in goldfish, ICV injections with GnRH2 not only stimulate spawning (Hoskins et al., 2008) but also decrease food intake (Hoskins et al., 2008; Matsuda et al., 2008) and hypothalamic orexin mRNA expression (Hoskins et al., 2008). Similarly, in zebrafish, ICV injections of GnRH2 decrease food intake (Nishiguchi et al., 2012). In addition, in goldfish, treatment with orexin stimulate feeding, inhibit spawning behavior, and decrease brain GnRH2 expression, suggesting a coordinated control of feeding and reproduction by the orexin and GnRH systems (Hoskins et al., 2008).

In winter flounder, fasting reduces both brain GnRH2 and GnRH3, but not GnRH1, mRNA expression levels (Tuziak and Volkoff, 2013b) and in zebrafish, GnRH2 brain mRNA levels increase in overfed fish (Nishiguchi et al., 2012). However, in Atlantic cod, neither GnRH2 nor GnRH3 brain transcripts are influenced by food deprivation (Tuziak and Volkoff, 2013a), suggesting that the role of GnRHs in the regulation of feeding might be species- and form-specific.

##### RFamides

RFamide peptides, first isolated in invertebrate species in the late 1970's and later found in vertebrates, act as neurotransmitters and neuromodulators. In vertebrates, the RFamide peptide family consists of PRL-releasing peptides (PrRP), PQRFamide peptides (neuropeptide FF, NPFF), pyroglutamylated RFamide peptide (QRFP)/26RFamides, LPXRFamide peptides (gonadotropin-inhibitory hormone, GnIH, in lower vertebrates, RFamide-related peptide-3, RFRP-3, in mammals) and kisspeptins (Tsutsui and Ubuka, 2013; Osugi et al., 2016). RFamides have been shown to regulate several physiological functions in vertebrates, including feeding (Bechtold and Luckman, 2007; Quillet et al., 2016). A number of RFamides have been identified in fish, although most have been examined for their role in reproduction and are not yet well characterized with regards to their potential role as feeding regulators.

In goldfish, IP or ICV administration of PrRP decrease food intake, and hypothalamic PrRP mRNA expression increases post-prandially and after food deprivation, suggesting an anorexigenic role for PrRP in goldfish (Kelly and Peter, 2006). In line with this hypothesis, in the euryhaline fish mudskipper (*Periophthalmus modestus*, Perciforme, gobidae), freshwater fish have lower food intake/growth rates than saltwater fish and higher brain and intestine PrRP mRNA expressions, suggesting that PrRP is involved in the regulation of feeding and energy homeostasis in this species (Sakamoto et al., 2002; Tachibana and Sakamoto, 2014).

Two neuropeptide FF receptor 1 (NPFFR1) genes have been identified in carp and shown to display variations in expression associated with growth-related traits (Peng et al., 2016). As NPFF1 is receptor for neuropeptide FF (NPFF) and the LPXRFamide peptide RFamide-related peptide (RFRP), which are involved in control of feeding behavior in both invertebrates and vertebrates, these data suggest that NPFFR1s might be related to the regulation of growth and body weight in common carp (Peng et al., 2016). Similarly, in seabass, LPXRFamide-ir cells and/or fibers are present in feeding, gustatory, sensory, and behavioral centers of the brain, suggesting that it could be involved in the regulation of foraging/feeding behavior (Paullada-Salmerón et al., 2016).

In goldfish, hypothalamic expression of 26RFa increases in fasted animals (Liu et al., 2009) and IP injections of human RFRP-3 decrease food intake (Mawhinney, 2007), indicating that these neuropeptides might regulate food intake and energy balance in cyprinid fish.

In sea bass, food-restricted male fish display an increase in both kisspeptin and kisspeptin receptor expressions in both pituitary and hypothalamus (Escobar et al., 2016), suggesting the kisspeptin system is affected by nutritional status. However, in goldfish, IP injections of mammalian kisspeptin appear to have no effect on feeding (Mawhinney, 2007).

#### CRF and the hypothalamus-pituitary-interrenal (HPI) axis

The major endocrine components of the hypothalamic–pituitary–adrenal (HPA) axis (or interrenal, HPI in lower vertebrates) are hypothalamic corticotropin-releasing factor (CRF, or corticotropin-releasing hormone, CRH), pituitary adrenocorticotropin (ACTH) and glucocorticoids (e.g., cortisol, corticosterone) from the adrenal/interrenal gland. CRF mediates the release of ACTH, which in turn stimulates the release of steroids by the adrenal/interrenal gland (Smith and Vale, 2006). The HPI axis regulates numerous physiological functions, including metabolic functions (e.g., blood glucose levels during fasting), food intake, reproduction, growth, and immunity. Urocortins (UCN) 1 (also termed urotensin 1 in fishes), 2, and 3 belong to a recently discovered family of CRF-related peptides, which functions are still not well characterized (Majzoub, 2006).

The role of the HPI axis in the regulation of feeding of fish has been examined in several fish species. In goldfish, ICV injections of CRF decrease feeding (De Pedro et al., 1993) and increase locomotor activity (Matsuda et al., 2013). In Ya fish, fasting decreases CRF brain expression levels (Wang et al., 2014) and goldfish exposed to the toxin fluoxetine have low food intake and increased brain expression of CRF (Mennigen et al., 2010), further suggesting an anorexigenic role for CRF in cyprinids. In goldfish, feeding fish with a diet containing low cortisol levels or implanting fish with cortisol-containing pellets result in higher food intake and CRF mRNA levels, compared to controls (Bernier et al., 2004). These results and others suggest that stress, cortisol and CRF can modulate food intake in Cypriniformes (Bernier et al., 2004).

In rainbow trout, CRF and urotensin 1 are anorexigenic, as ICV injections of either peptides inhibit feeding (Ortega et al., 2013). In addition, hypoxia stress suppresses appetite and increases forebrain CRF and urotensin mRNA levels, suggesting that, in Salmoniformes, CRF-related peptides might mediate the hypoxia-induced reduction in food intake (Bernier and Craig, 2005).

In Siberian sturgeon, IP injections of urocortin 3 inhibit feeding, and UCN3 brain mRNA expression levels increase post-feeding and decrease during fasting, suggesting that UCN3 acts as a satiety/anorexigenic factor in fish (Zhang et al., 2016c).

For more extensive reviews on the regulation of feeding by the HPI, please refer to previously published works, including (Bernier and Peter, 2001; Bernier, 2006; Flik et al., 2006; Lowry and Moore, 2006; Backström and Winberg, 2013).

### “novel” appetite-regulating peptides

#### Amylin

Amylin (or islet amyloid polypeptide, IAPP), a hormone co-secreted with insulin from pancreatic β-cells, inhibits feeding in mammals (Riediger et al., 2003). In fish, the role of amylin in feeding has only been examined in goldfish. In this species, IP or ICV amylin treatments decrease food intake whereas ICV injections of an amylin receptor antagonist (AC 187) stimulate feeding (Thavanathan and Volkoff, 2006), suggesting an anorexigenic role for amylin in fish.

#### Apelin

Apelin is a peptide first identified in bovine stomach as a ligand for the orphan receptor APJ, with close identity to the angiotensin II (Ang II) receptor (Tatemoto et al., 1998; Habata et al., 1999) and subsequently shown to be involved in multiple physiological processes (see O'Carroll et al., 2013, for review) including feeding and metabolism in mammals: for example, apelin injections decrease food intake (O'Shea et al., 2003), and in adipocytes, apelin expression is inhibited by fasting (Boucher et al., 2005) and its secretion is regulated by insulin (Boucher et al., 2005).

In fish, apelin appear to be orexigenic: apelin injections increase food intake in goldfish (Volkoff and Wyatt, 2009) and cavefish (Penney and Volkoff, 2014). Fasting induces increases in brain apelin mRNA expression in Ya-fish (Lin et al., 2014a) and red-bellied piranha (Volkoff, 2014a). Moreover, in goldfish, the obesogen factor tributyltin (TBT) stimulates food intake and also increases brain apelin expression (Zhang et al., 2016b). In cavefish, IP injections of apelin increase orexin brain expression, and CCK injections induce a decrease in brain apelin expression (Penney and Volkoff, 2014), an indication that apelin interacts with other appetite regulators. Similarly, brain injections of the anorexigenic factor spexin reduce apelin brain expression (Wong et al., 2013) and *in vitro* treatment of brain fragments with apelin increase expressions of orexigenic peptides—i.e., orexin—and decrease CART expression (Volkoff, 2014b). Overall, the data suggest an orexigenic role for apelin in Cypriniformes. In cunner (Perciforme), summer fasting decreases intestinal apelin mRNA levels (Hayes and Volkoff, 2014), suggesting that GIT apelin might not be involved in the regulation of feeding. In common carp- but not in trout barb *Capoeta trutta*-, there is a negative correlation between apelin levels and body weight (Köprücü and Algül, 2015), suggesting that apelin might not be involved in metabolic processes leading to weight gain in some species.

#### Arginine vasotocin

Arginine vasotocin (AVT) is the mammalian homolog of arginine vasopressin (AVP), and has been shown to have diverse and complex roles in fish physiology, including regulation of metabolic processes, stress responses and several behaviors (Balment et al., 2006). In rainbow trout, AVT treatments decrease feeding, and increase plasma levels of cortisol and glucose, brain serotonergic activity, and hypothalamic levels of POMC and CART, suggesting it acts as an anorexigenic factor in fish (Gesto et al., 2014).

#### Endocanabinoid system

In mammals, the endocannabinoid system (ECS), which consists of cannabinoid receptors (CB1 and CB2) and endogenous cannabinoids, is involved in the regulation of several physiological functions, including feeding and energy balance (Pagotto et al., 2006).

In goldfish, CB1 and CB2 are both expressed in brain, where CB1 co-localizes with NPY (Cottone et al., 2013). Treatment with low doses of the endocannabinoid receptor agonist anandamide (AEA) increases food intake (Valenti et al., 2005), and food deprivation increases CB1 and AEA brain mRNA levels (Cottone et al., 2009), suggesting the involvement of the ECS in the control of energy intake in Cypriniforme. Similarly, in sea bream *Sparus aurata* (Perciforme), AEA administered via water increases food intake and NPY brain mRNA levels (Piccinetti et al., 2010). In common sole, *Solea solea* (Pleuronectiforme), feeding fish with dietary nucleotides reduce CB1 brain transcript levels, suggesting that feeding and diets modulate the ECS (Palermo et al., 2013).

#### Nesfatin-1

Nesfatin-1, discovered in 2006 in mammals, is a peptide secreted from hypothalamic nuclei related to appetite regulation, from the precursor non-esterified fatty acid/nucleobinding 2 (NUCB2), and has been shown to reduce feeding and water intake in mammals (Ayada et al., 2015). In fish, the role of nesfatin-1 as an appetite regulator has been examined in Cypriniformes and Salmoniformes.

In goldfish, nesfatin-1 has been shown to be involved in the regulation of feeding and metabolism: nesfatin-1-like and ghrelin-like ir co-localize in both enteroendocrine and hypothalamic cells; IP or ICV injections of nesfatin-1 inhibit both food intake and brain expressions of ghrelin and NUCB2; and fasting increases both hepatic and hypothalamic NUCB2 mRNA levels (Gonzalez et al., 2010; Kerbel and Unniappan, 2012). In addition, NUCB2 mRNA levels increase in liver and hypothalamus in fish fed fat-enriched diets and decrease in gut after long-term feeding with a high-protein diet, suggesting that macronutrients regulate the expression of NUCB2/nesfatin-1 (Blanco et al., 2016a). In zebrafish, two isoforms of NUCB2 (NUCB2A and NUCB2B) exist, and both mRNAs decrease in the brain post-prandially and after food deprivation, suggesting an anorexigenic role for nesfatin-1 (Hatef et al., 2015). In Ya-fish, NUCB2A mRNA levels increase post-prandially in both hypothalamus and intestine, and fasting induces a decrease in NUCB2A mRNA levels in the hypothalamus, but an increase in the hepatopancreas, suggesting anorexigenic and metabolic roles (Lin et al., 2014b). However, in rainbow trout (*Oncorhynchus mykiss*), plasma nesfatin-1 levels are similar between fed and fasted females (Caldwell et al., 2014).

#### Neuropeptide B, neuromedin S, and neuromedin U

Neuropepide B (NPB), and neuromedins S (NMS) and U (NMU) are newly discovered mammalian short peptides that have been shown to affect feeding in fish.

NPB has been characterized in Nile tilapia (Perciforme), where it is expressed in brain and spinal cord. In this species, fasting increases NPB brain mRNA expression, and IP injections of NPB increase brain mRNA expression of NPY and CCK and inhibit pituitary GH expression, suggesting NPB is involved in feeding and growth in fish (Yang et al., 2014).

In both zebrafish (Chen et al., 2016a) and orange-spotted grouper (Li et al., 2015), an NMS-related protein (NMS-RP) has been identified that appears to act as an orexigenic factor. In both species, IP administration of species-specific NMS-RP increases both NPY and orexin expressions, and hypothalamic levels of NMS mRNA increase after food deprivation.

NMU has been characterized in Cypriniformes (carp, goldfish) and Perciformes (orange-spotted grouper). In both common carp (Kono et al., 2012) and goldfish (Maruyama et al., 2008), several forms of NMU (3–5) have been isolated and their mRNA expressions shown to decrease upon fasting, suggesting a role in feeding and metabolism (Kono et al., 2012). Similarly, in orange-spotted grouper, hypothalamic NMU mRNA levels decrease in fasted fish and increase post-feeding (Li et al., 2015), suggesting an anorexigenic role. In goldfish, central injections of NMU inhibit feeding and locomotor behaviors (Maruyama et al., 2008) and increase brain CRF mRNA expression levels (Maruyama et al., 2009) and in grouper, IP injections of NMU down-regulate hypothalamic NPY expression (Li et al., 2015), suggesting that the anorexigenic actions of NMU are mediated by the CRF system and an inhibition of the NPY system.

#### Obestatin

Obestatin, a gastrointestinal peptide discovered in 2005, is derived from the same precursor as ghrelin and inhibits food intake in mammals (Cowan et al., 2016). In grass carp, although IP injections of an obestatin-like peptide alone do not affect food intake or the expression levels of NPY, CART, or POMC, when co-injected with ghrelin, it blocks ghrelin-induced stimulation of appetite and up-regulation of expressions of NPY and NPY receptors (Yuan et al., 2015), suggesting that obestatin might inhibit the ghrelin system in Cypriniformes.

#### Octadecaneuropeptide

The octadecaneuropeptide (ODN) is a peptide belonging to the family of endozepines and is generated through the cleavage of diazepam-binding inhibitor (DBI) in the mammalian central nervous system (CNS) (Tonon et al., 2006). ODN acts as an inverse agonist of central-type benzodiazepine receptors (CBR) and inhibits food intake in rodents (do Rego et al., 2006).

Immunocytochemical methods first showed the presence in brain and pituitary of rainbow trout (Malagon et al., 1992) and more recently in the agnathan Atlantic hagfish, *Myxine glutinosa* (Myxiniforme, myxinidae; Candiani et al., 2000). Central injections of goldfish ODN inhibit food intake (Matsuda et al., 2007b) and stimulate locomotor activity (Matsuda et al., 2011b), and increase POMC brain mRNA levels (Matsuda et al., 2010), suggesting that the anorexigenic actions of ODN are in part mediated by the melanocortin system.

#### Pituitary adenylate cyclase activating polypeptide (PACAP)

Originally identified in the ovine hypothalamus (Miyata et al., 1989), pituitary adenylate cyclase-activating polypeptide (PACAP) belongs to the secretin/glucagon family of peptides that also includes secretin, glucagon, glucagon-like peptides and vasoactive intestinal peptide (Sherwood et al., 2000). In rodents, central injections of PACAP decrease food intake (Morley et al., 1992).

PACAP has been cloned in several fish, including Anguilliformes European eel (*Anguilla anguilla*) (Montero et al., 1998), Cypriniformes (e.g., zebrafish Sherwood et al., 2007, goldfish Matsuda et al., 1997), Gadiformes (cod Xu and Volkoff, 2009), Pleuronectiformes (e.g., olive flounder Nam et al., 2013), Salmoniformes (e.g., Atlantic salmon Parker et al., 1993), and Siluriformes (Thai catfish *Clarias macrocephalus* McRory et al., 1995, darkbarbel catfish *Pelteobagrus vachelli* Xu et al., 2012) as well as elasmobranchs (e.g., marbled electric ray *Torpedo marmorata* Agnese et al., 2016, stingray *Dasyatis akajei* Matsuda et al., 1998). In several fish, PACAP stimulates GH secretion by pituitary cells (see review in Gahete et al., 2009), but its role in regulating feeding is still unclear. In goldfish, central or peripheral PACAP injections inhibit food intake (Matsuda et al., 2005) and locomotor activity (Matsuda et al., 2006) and these actions might be mediated in part by the stimulation of POMC and CRH pathways (Matsuda and Maruyama, 2007). Similarly, in grass carp, central NPY injections decrease brain PACAP expression (Zhou et al., 2013), suggesting an anorexigenic role for PACAP in Cypriniformes. In Atlantic cod, PACAP inhibits intestinal smooth muscle contractions (Olsson and Holmgren, 2000), and although brain expression levels are not affected by 30 days of food deprivation, they increase after during the re-feeding period (Xu and Volkoff, 2009), suggesting that PACAP is involved in the regulation of feeding and digestive processes (Xu and Volkoff, 2009).

#### Secretoneurin

Secretoneurin (SN) is a short peptide derived from a secretogranin-II (SgII, also called chromogranin C) precursor protein (Zhao et al., 2009). In goldfish, ICV injections of the SN increase food intake and locomotor behavior (Trudeau et al., 2012), increase mRNA levels of hypothalamic NPY and decrease hypothalamic CART. In addition, fasting increases telencephalon SgII mRNA levels (Mikwar et al., 2016), suggesting that, in fish, SN might act as an orexigenic factor.

#### Spexin

Spexin (SPX) is a peptide identified in 2007 in mammalian adipose tissue. SPX expression is down-regulated in obese humans and rats, and subcutaneous injections of SPX reduce food intake and increase locomotion (Walewski et al., 2014).

In goldfish, SPX appear to act as an anorexigenic factor: brain injections of SPX inhibit both basal and NPY- or orexin-induced food consumption, decrease brain expressions of orexigenic factors (NPY, AgRP, and apelin) and increase that of anorexigenic factors (CCK, CART, POMC, MCH, and CRH), and brain SPX mRNA levels increase post-prandially (Wong et al., 2013). Similarly, in the orange-spotted grouper, IP administration of SPX increases hypothalamic mRNA levels of POMC and inhibits orexin expression, suggesting an anorexigenic role (Li S. et al., 2016). However, grouper SPX hypothalamic expression increases following long-term food deprivation (Li S. et al., 2016), suggesting that spexin might be a short-term satiety factor rather than a long-term hunger signal.

## Concluding remarks

Although the basic mechanisms regulating feeding seem to be relatively conserved between mammals and fish, it must be kept in mind that major physiological differences exist between these two groups. Fish are ectotherms and thus have lower metabolic rates than mammals and more sensitive to environmental changes, their physiology changing with their fluctuating surroundings. They also have different means of energy/nutrient storage (e.g., fat storage in liver rather than subcutaneous adipose tissue), and different growth patterns (as opposed to mammals, fish continue to grow after sexual maturity), suggesting that the endocrine regulation of energy balance, feeding and growth in fish differs from that of mammals.

Comparative studies at the genome level have revealed conserved sequences for appetite regulators across mammalian and fish species, indicating potentially conserved biological functions. Whereas the genome of all vertebrates is the result of two rounds (2R) of whole genome duplication (WGD) occurring in early vertebrate evolution, additional WGDs occurred in the teleost fish ancestor (3R) and most recently in certain teleost lineages (4R, e.g., salmonidae and cyprinidae), leading to the presence of increased gene copy numbers and multiple protein isoforms with potentially different physiological functions (Glasauer and Neuhauss, 2014), making the fish model potentially more complex. One must thus keep in mind that fish feeding-regulating hormones might not always have the same function as their mammalian homologs.

Fish are an extremely diversified group, with a great variability in feeding habits and requirements as well gut morphology and digestion processes. Fish can be carnivores, herbivores, omnivores or detritivores, with different feeding habits often seen within the same family (e.g., herbivore Mbuna cichlids and carnivore Nile tilapia in cichlidae; herbivore/omnivore pacu and carnivore piranha in serrasalmidae). Different fish species not only require different compositions of food, but also different amounts of food and feeding frequencies (Moore, 1941). Diet and feeding habits is reflected in the anatomy and physiology of the gastrointestinal tract. For example, carnivores or omnivores (such as most Characiformes and Siluriformes) have stomachs, pyloric caeca, and relatively short and straight intestines, whereas herbivores or detrivores (e.g., Cypriniformes and Cyprinodontiformes) may lack both stomach and caeca and have long and convoluted intestines (Leknes, 2015). Different diets and guts translate into different digestive enzyme profiles and different methods of nutrient storage (Day et al., 2011), as seen for lipids (e.g., in muscle in “oily” fish such as salmon and herring vs. liver in “lean” fish such as cod and flatfish), which usage might also be affected by reproductive stages and modes (guarding vs. non guarding; mature vs. immature; oviparous vs. viviparous).

Given the high diversity within fish, one should thus be careful when generalizing results from one species to all fish. Comparative studies establishing similarities and differences among species should be valuable to understand mechanisms regulating feeding. However, the large number of species poses the problem of the model species to choose. To date, most studies examining the neuroendocrine regulation of fish still use “classical” model species, i.e., cyprinids and salmonids. These somewhat differ from most fishes, as they display polyploidy, and might not represent a “perfect” model, but they are easily available and maintained, as their different holding conditions, habitats and diets, are well known. However, new species, in particular commercially important aquaculture species such as Perciformes (the largest teleost order) and Pleuronectiformes have recently been examined.

The increasing number of studies and species examined often generates conflicting and sometimes contradictory results. This variability might express true differences between species, but contradictory data also occur within same species. This variability might have several reasons. First, there is a great variability in the nature and nomenclature of isoforms examined (e.g., within CART forms). Second, when comparing studies, it is sometimes difficult to compare results obtained using different protocols (e.g., different lengths of fasting) and techniques (e.g., mRNA vs. protein vs. plasma levels), in particular because changes in gene expression do not necessarily translate into different protein levels or circulating levels. Finally, fish used between studies are often of different ages (e.g., larval vs. adult), sexual maturity (immature vs. mature spawning or non-spawning) or even environmental conditions (e.g., temperatures, photoperiods), all of these factors influencing feeding.

Even in mammals, the regulation of appetite is not yet fully understood. Using a comparative approach involving multiple fish species, perhaps choosing representative families/species from each fish group, and complementary methods might help us start drawing accurate models for the endocrine regulation of feeding in fish.

## Author contributions

HV designed this review, including table and figure, researched, acquired and analyzed all the information, drafted and revised the manuscript, and approved the version to be published. To HV's knowledge, information contained in this review and studies cited within it have been appropriately checked for accuracy or integrity.

### Conflict of interest statement

The author declares that the research was conducted in the absence of any commercial or financial relationships that could be construed as a potential conflict of interest.
